# Production and characterization of two medium-chain-length polydroxyalkanoates by engineered strains of *Yarrowia lipolytica*

**DOI:** 10.1186/s12934-019-1140-y

**Published:** 2019-05-31

**Authors:** Coraline Rigouin, Sophie Lajus, Connie Ocando, Vinciane Borsenberger, Jean Marc Nicaud, Alain Marty, Luc Avérous, Florence Bordes

**Affiliations:** 1 LISBP, CNRS, INRA, INSA, Université de Toulouse, Toulouse, France; 2 BioTeam/ICPEES-ECPM, UMR CNRS 7515, Université de Strasbourg, 25 rue Becquerel, 67087 Strasbourg Cedex 2, France; 3 Micalis Institute, INRA-AgroParisTech, UMR1319, Team BIMLip: Integrative Metabolism of Microbial Lipids, Domaine de Vilvert, 78352 Jouy-en-Josas, France; 4 Carbios - Biopôle Clermont-Limagne, 3 rue Emile Duclaux, 63360 Saint-Beauzire, France

**Keywords:** Medium-chain-length polyhydroxyalkanoate, *Yarrowia lipolytica*, Bioproduction, Engineering, Biopolymer characterization

## Abstract

**Background:**

The oleaginous yeast *Yarrowia lipolytica* is an organism of choice for the tailored production of various compounds such as biofuels or biopolymers. When properly engineered, it is capable of producing medium-chain-length polyhydroxyalkanoate (mcl-PHA), a biobased and biodegradable polymer that can be used as bioplastics or biopolymers for environmental and biomedical applications.

**Results:**

This study describes the bioproduction and the main properties of two different mcl-PHA polymers. We generated by metabolic engineering, strains of *Y*. *lipolytica* capable of accumulating more than 25% (g/g) of mcl-PHA polymers. Depending of the strain genetic background and the culture conditions, we produced (i) a mcl-PHA homopolymer of 3-hydroxydodecanoic acids, with a mass-average molar mass (M_w_) of 316,000 g/mol, showing soft thermoplastic properties with potential applications in packaging and (ii) a mcl-PHA copolymer made of 3-hydroxyoctanoic (3HO), decanoic (3HD), dodecanoic (3HDD) and tetradecanoic (3TD) acids with a M_w_ of 128,000 g/mol, behaving like a thermoplastic elastomer with potential applications in biomedical material.

**Conclusion:**

The ability to engineer *Y. lipolytica* to produce tailored PHAs together with the range of possible applications regarding their biophysical and mechanical properties opens new perspectives in the field of PHA bioproduction.

**Electronic supplementary material:**

The online version of this article (10.1186/s12934-019-1140-y) contains supplementary material, which is available to authorized users.

## Background

Polyhydroxyalkanoates (PHAs) are a family of microbial, biobased and biodegradable polyesters representing an attractive ecofriendly alternative to some fossil-based polymers. They are biosynthesized by various types of microorganisms in conditions of excess of available carbon and of a limited supply of one of the nutrients essential to bacterial cell growth [1]. The biopolymers are synthesized by polymerization of 3-hydroxy fatty acids (3-OH FA) by a PHA synthase (PhaC), inside the bacterial cell in the form of granules that serve as carbon and energy storage compounds [2]. PHA polymers are categorized into subclasses according to the side chain of their monomers: in particular, short-chain-length PHAs (scl-PHA with three to five carbon monomers) and the medium-chain-length PHAs (mcl-PHA with six to fourteen carbon monomers). Copolymers of scl- and mcl-PHA, block copolymers PHA and homopolymers PHA are named based on the monomer arrangements in the polymer chains [3]. The properties of these biopolymers depend on their molar masses and their macromolecular architectures (the side chains of their monomers), [3, 4]. For example, the well-studied scl-PHA, P3HB (poly-3-hydroxybutyrate), has poor mechanical properties. Indeed, the isotactic and linear chains of P3HB result in the formation of large spherulites during crystallization, which may lead to a highly crystalline (60%) and consequently a brittle material. Besides, P3HB presents a poor processability with thermal input due to a high thermal sensitivity at a temperature higher than the melting temperature, with strong chains scissions. P3HB and PHBV (poly(3-hydroxybutyrate-*co*-3-hydroxyvalerate)) with a low HV content have not been largely utilized in biomedical areas due to the stiff and brittle nature. On the other hand, mcl-PHA properties ranges from rubber to tacky polymers with higher thermal resistance [5, 6]. Diverse products can thus be obtained from PHA including bioplastics, chemicals and feed supplements [7]. Besides, different applications in medical and pharmaceutical industries can be considered [8, 9]. The main drawback to PHA usage is its high cost of production compared to conventional fossil-based polymers. Nonetheless, advances in PHA research have shown tremendous progresses in (i) finding alternative carbon sources for PHA production (ii) controlling PHA processing during production, and (iii) controlling PHA composition and molar mass [10–12].

Medium-chain-length PHAs are made up of monomer units with 6–14 carbon atoms and were first identified in *Pseudomonas putida* GPo1 [13]. mcl-PHA are interesting as they can bear different functional groups in the side chains that can modify the physico-chemical and physical properties, offering a wide range of applications [3]. Depending of the monomer composition, mcl-PHA have been demonstrated to have the elastomeric properties required for specific applications such as packaging materials or biomedical applications [3, 14]. Nevertheless, inconsistencies on their molar masses and their structures lead to variations of their thermo-mechanical properties, which are a strong drawback for their uses. Some organisms are naturally capable of synthesizing mcl-PHA and have been engineered in order to enhance polymer production or to produce mcl-PHA of interest [15–17]. Non-natural mcl-PHA producers have also been engineered such as yeast [18–21], bacteria [16, 22–24], and plants [23, 25].

*Yarrowia lipolytica* is a GRAS organism (Generally Regarded As Safe) with large potentials in industrial biotechnology [26]. This yeast is naturally capable of producing and accumulating large amount of lipids (more than 50% of its dry weight in large-scale fermentations [27]). In regard to such potential, this yeast is becoming an organism of choice for the production of many compounds such as lipids [27, 28], proteins [29] and biopolymers [18]. It is naturally capable of growing on different substrates (sugars, oils, alkane, glycerol) and has been recently engineered to accept inexpensive carbon sources [30, 31]. *Y. lipolytica* was used to produce both PHA copolymer and homopolymer with an external fatty acid (FA) supply and the expression of the PhaC synthase from *Pseudomonas aeruginosa* [18, 19]. Haddouche et al. [19] redirected the FA flux toward β-oxidation by deleting the neutral lipid synthesis pathway and by overexpressing the 2-enoyl-CoA hydratase domain of the MFE protein to enhance the synthesis of the 3-OH FA precursor. This led to an accumulation of PHA up to 7% of cell dry weight (CDW). However, the properties of the produced PHA were not investigated, likely because of its low accumulation level.

The production of tailor-made mcl-PHAs using diverse FA precursors could lead to different polymer properties for new materials. When using an heterologous organism, it is important to evaluate the resulting polymer with respect to the accumulation capacity, the yield, the polymer constituents, and the material properties. In this paper, we describe the production and the characterization of two mcl-PHA in *Y. lipolytica*, an homopolymer and a copolymer, highlighting the capacity of the engineered strain to accumulate high amount of polymer and giving evidence for the bioproduction of two different materials with distinct properties.

## Methods

### Strains construction

PhaC variants strains: the wild type version of PhaC was cloned on the JMP62 pTEF_URA3ex plasmid [32]. Variants of the PhaC synthase were then generated by directed mutagenesis using the QuikChange II Site-Directed Mutagenesis Kit (Agilent), and the list of primers listed in Additional file 1: Table S4. After directed mutagenesis, the gene sequences were checked by Sanger sequencing services from GATC Biotech (Constance, Germany). After construction, all the plasmids were linearized and used to transform *Y. lipolytica* strain JMY_1877 [33]. The yeast cells were made competent and transformed using Frozen-EZ Transformation kit (Zymo Research, Irvine, CA, USA). Yeast transformants were selected by auxotrophy on the adequate minimal medium and the presence of the genes of interest was checked by PCR. All the constructed strains used are described (Additional file 1: Table S1). ThYl_1166 was constructed by transformation of the strain JMY2333, a derivative of the strain JMY1915 [19], with the plasmids JMP62 pTEF-PhaC_E130D S325T S477R-URA3ex and the plasmid JMP62 pTEF-CpPCT-LEU2ex. ThYl_657 was obtained by transformation of a the strain JMY2475 with the plasmid JMP62 pTEF-PhaC_E130D S325T S477R-URA3ex and the strain ThYl_1024 by transformation of the strain JMY2475 with the plasmid JMP62 4UAS-tef MfeC-LEU2ex [34] and the plasmid JMP62 pTEF-PhaC_E130D S325T S477R-URA3ex.

### Cultures for PHA production

Pre-cultures were started from fresh colonies in 20 mL of the rich medium YPD (yeast extract 10 g/L, bactopeptone 10 g/L, glucose 10 g/L). Minimal medium YNB (glucose 40 g/L, YNB w/o AA 1.7 g/L, NH_4_Cl 5 g/L, phosphate buffer pH 6.8 50 mM) was used to grow cells for PHA production. Solutions of fatty acid methyl esters [methyl myristate (mC14) and methyl laurate (mC12)] were prepared at 20% (v/v) in H_2_O and Tergitol or tween. After 24 h and 48 h of culture, 2.5 g/L mC14 or mC12 was added. As exogenous medium chain fatty acids may be toxic to the cells at high concentration, mC14 was added sequentially and during the exponential growth phase rather than in one time at the beginning. After 5 days of culture, cells were harvested by centrifugation (500×*g* 10 min), washed once with 50% isopropanol (to remove excess fatty acids from the medium) and twice with H_2_O and stored at − 80 °C. Upon thawing, cells were resuspended in 100 mM Tris–HCl buffer at pH 8 with 0.5 mg/mL of zymolyase and lysed with a 28 °C incubation for 18 h with shaking. Cells were then freeze-dried until PHA extraction.

### PHA extraction

Polymer was extracted with chloroform from the dried cells using a Soxhlet apparatus. Typically, 50 mL of solvent were used to extract polymer from 1.5 g of dried cells and the filling and emptying cycles of the chamber were allowed to process 10 times before collection of the solvent containing the extracted materials. PHA in chloroform was then filtered using a 40 mL Graces’ Reveleris^®^ cartridge and precipitated by addition of 1 volume of ethanol for 2 volumes of chloroform, followed by cooling at − 20 °C. After centrifugation, the supernatant was discarded, the PHA was dried on air and then used for further analysis.

### NMR

Polymer quantities were determined by NMR on a Bruker Avance II 500 spectrometer. The cells extracts were thoroughly dried, prior to being diluted in CDCl_3_ containing 1% TMS (internal standard) and transferred to 5 mm NMR tubes. NMR spectra were recorded at 298 K. Each NMR spectrum was acquired using an excitation flip angle of 30° at a radiofrequency field of 29.7 kHz, a relaxation delay of 10 s and 2 dummy scans. For each experiment, 16 scans were performed with a repetition delay of 6.5 s. PHA concentrations were determined by integration of the two specific AB double doublets at 2.55 ppm.

### Transmethylation and GC/MS analysis

PHA monomer composition was analyzed by transmethylation of the polymer in hot acid methanol [35]. Briefly, 2 mL of a solution of methanol (with C17 FA prepared at 0.2 mg/mL as standard) with 2.5% sulfuric acid was added to the dried sample in addition to 1 mL of Toluene. Samples were heated at 80 °C for 3 h. Once the samples were cooled down, biphasic liquid extraction took place using 1.5 mL of 0.5 M NaCl and 1.5 mL hexane (containing mC20 FA at 0.1 mg/mL as internal standard). Analyses were performed on the organic phase with a gas chromatography coupled with mass spectrometry (GC–MS) *TRACE*™ 1310 equipped with the *TRACE*™ TR-5 column (Thermo-scientific). Standards of 3-OH fatty acids methyl esters were used to determine retention time and for relative quantification.

### Size exclusion chromatography (SEC)

The number average molar mass (*M*_n_), the mass average molar mass (*M*_w_) and the dispersity (*Đ*) of the PHA samples were determined by SEC, using a Malvern Instrument apparatus (Viscotek RImax). This device was equipped with a guard column 10 mm (8 μm) and three 300 mm columns (50, 150 and 500 Å). Refractive index (RI) and ultra-violet (UV) detectors were used. Tetrahydrofuran (THF) was used as the eluent at a flow rate of 1 mL/min. The apparatus was calibrated with linear polystyrene standard from 162 to 20,000 g/mol.

### Elemental analysis

Elementary analyses were performed on a ThermoFisher Scientific “Flash 2000” (US) device (absolute precision of 0.3%) with 1 mg sample burned up to 950 °C.

### Thermogravimetric analysis (TGA)

Thermal degradations were studied by TGA. Measurements were conducted under nitrogen atmosphere (flow rate of 25 mL/min) using a Hi-Res TGA Q5000 apparatus from TA Instruments. Samples (1–3 mg) were heated from room temperature up to 700 °C at a rate of 20 °C/min.

### Differential scanning calorimetry (DSC)

Crystallization behavior of both PHAs was studied on a DSC Q 200 (TA Instruments). Samples of around 3 mg were heated from 25 to 180 °C at 10 °C/min under nitrogen flow (50 mL/min). This first scan was conducted in order to erase the thermal history of the bulk polymer. In ramp 2, the sample was cooled at 5 °C/min to − 70 °C and kept isothermal for 3 min. In ramp 3, the sample was once again heated at 10 °C/min to 180 °C. Ramp 3 was used for determination the glass transition temperature (T_g_), melting temperature (T_m_) and heat of fusion (ΔH_m_).

### Uniaxial tensile tests

After a thermal treatment at 37 °C for 5 days (body temperature) of thin films (10 × 5 × 0.2 mm^3^) prepared by solvent casting, tensile tests were performed with a Discovery HR hybrid rheometer (TA instruments) at a crosshead displacement rate of 10 mm/min, at room temperature.

## Results and discussion

### Polymer accumulation depending on PhaC synthase variant expressed by the yeast

Recent studies have demonstrated that some specific mutations in PhaC can enhance, alone or in combination, PHA polymer production and composition when expressed in bacteria [36, 37]. Consequently, we investigated the capacity of different PhaC synthase variants (Additional file 1: Table S1) to produce and accumulate PHA copolymer in *Y. lipolytica*. The strain JMY_1877 [19] was used as a chassis strain to express the PhaC variants under the strong constitutive promoter pTEF and to produce PHA copolymer. PhaC was targeted to the peroxisome as this compartment is the site of fatty acid degradation by the β-oxidation pathway and therefore the site where 3-OH FAs, the substrates of PhaC, are synthesized as intermediates of the β-oxidation pathway. In this strain, lipid accumulation is abolished by the deletion of acyl-transferases in order to reduce fatty acids storage and improve their availability for PHA production.

The strains expressing the different PhaC variants were grown for PHA production as described in “Methods” section with methyl myristate (mC14) as precursor. Cells were then harvested, the polymer extracted and the polymer accumulation and composition were measured (Additional file 1: Table S2). The first set of strains expressing PHA variants that we generated (ThYl_1475, 1479, 1480, 1481, 1485, 1487 and 1494) accumulated higher or equivalent levels of polymer than the ThYl_1475 strain expressing the wild-type PhaC. The most interesting variant, ThYl_1494, combined the four mutations E130D S325T S477R Q481M and led to 27% accumulation of polymer. The second set of variants generated (corresponding to the strains ThYl_1491, 1496 and 1498) combined the quadruple mutations and the mutations S482G, L484V and A547V respectively. No synergistic effect on polymer accumulation was observed: the strain ThYl_1491 showed similar performance than ThYl_1494 and the strains ThYl_1496 and ThYl_1498 lost their ability to improve polymer accumulation. Finally, we chose to use the quadruple variant PhaC E130D S325T S477R Q481M for downstream experiments.

NMR analysis and transmethylation followed by GC–MS analysis gave evidence for the production of a PHA composed of 3-OH FAs of different chain lengths, except for the strain ThYl_1496 in which no production occurred (Additional file 1: Table S2). In term of fatty acid composition, no major difference was observed among the PHA produced by the different strains tested. The composition is as follows: 26 to 30% of 3HO; 32 to 36% of 3HD; 25 to 29% of 3HDD and 9 to 13% of 3HTD.

### mcl-PHA copolymer production

Having identified a PhaC variant with a high capacity to accumulate PHA polymers in *Y. lipolytica*, we decided to investigate the physical properties of two polymers produced by this yeast: a copolymer and an homopolymer.

For the copolymer production, we used the strain ThYl_1166 (Additional file 1: Table S1). In this strain the MFE protein [containing the two hydrogenase domains (domains A and B) and the single hydratase domain (domain C)] is expressed under pPOX2, the strong fatty acid inducible promoter, to force fatty acid degradation. Consequently, this strain generates high amounts of 3-OH FAs, the substrates of PhaC synthase. This host successfully expressed the variant PhaC_E130D S325T S477R Q481M. ThYl_1166 was grown as described in “Methods” section with mC14 FA. After 5 days, when the OD reached 25 and the glucose was completely consumed, we stopped the cultures. After polymer extraction, NMR and transmethylation analysis revealed that in these conditions, the strain ThYl_1166 accumulated 27% (g/g of DCW) of a mcl-PHA polymer composed of 29% of 3HO, 35% of 3HD, 26% of 3HDD and 10% of 3HTD (Table 1 and Additional file 1: Figure S1). These results are quite closed to the one observed for the PHA produced by strain ThYl_1491 suggesting that the difference in genetic pattern between ThYl_1166 (Q4, Δmfe1, pPOX-MFEABC, pTEF-PhaC_E130D S325T S477R Q481M-URA3ex, pTEF-CpPCT-Leu2ex) and ThYl_1491 (Q4, pTEF-PhaC_E130D S325T S477R Q481M S482G L484V-URA3ex, Leu2ex) did not affect PHA composition and accumulation.

**Table 1:** PHA accumulations and compositions

Strain	Substrate	PHA accumulation % (g/g)	Fraction of 3OH FA (mmol)			
			3HO	3HD	3HDD	3HTD
ThYl_1166	mC14 (5 g/L)	27%	29%	35%	26%	10%
ThYl_1024	mC12 (5 g/L)	28%	nd	nd	> 99%	nd

*nd* not detectable

### mcl-PHA homopolymer

For the homopolymer production, only the C domain of the MFE protein (MFEC), encoding the 2-enoyl-CoA hydratase, essential to generate exclusively the 3-OH FA, substrates of PHA synthase was re-introduced into a Q4 Δ*mfe* strain expressing the quadruple variant of the PhaC synthase (E130D, S325T, S477R and Q481M). The two dehydrogenase domains of the MFE protein were not re-introduced in order to avoid further degradation of the 3-OH FAs. We obtained the strain ThYl_657 (Additional file 1: Table S1). To optimize the production, we also generated a strain overexpressing MFEC by adding in the genome, a second copy of the mfeC gene under the control of the strong promoter 4UASTEF. We obtained the strain ThYl_1024 (Additional file 1: Table S1). To evaluate the best substrate for homopolymer production, both strains ThYl_1024 and ThYl_657 were first grown in minimal medium with either methyl laurate (mC12) or mC14 as fatty acid precursors. After 5 days of culture, polymers were extracted and the polymers accumulations were estimated by NMR. The best accumulation level was obtained with the strain ThYl_1024 grown with mC12 with 15% (g/g) vs. less than 9% (g/g) for the others conditions (Additional file 1: Table S3). This result highlighted the interest to have a double copy of the mfeC gene to maximize the pool of the 3-OH FA precursor. Therefore, this combination of strain and fatty acid was used for a larger scale production.

ThYl_1024 was grown in 10 L of YNB supplemented with mC12 FA. After 5 days of culture and the addition of twice 2.5 g/L of mC12 the polymer was extracted and analyzed. In these conditions, ThYl_1024 strain accumulated 28% of PHA. NMR studies gave evidence of a homopolyester and GC analysis confirmed the production of a polymer composed of 3HDD chains (Additional file 1: Figure S2 and Table 1).

This study clearly validates the optimization of the bioproduction of two types of polymers: by testing PhaC variants, we identified a PhaC enzyme variant (with E130D S325T S477R and Q481M mutations) with high performance in polymer accumulation in *Y. lipolytica.* We showed that the four mutations introduced within PhaC led to a fourfold improvement of polymer accumulation in the yeast with no impact on cell growth and polymer composition. It is quite surprising that the side chains of the 3-OH-FAs composing the PHA produced by the PhaC variants were not smaller than the ones composing the polymer produced by the wild-type PhaC. Indeed, some of the mutations tested were described to preferentially accommodate 3-hydroxy-butyric acid substrate (with four carbon atoms only) [37, 38]. Our results suggest that those mutations either improved the PhaC efficiency in the yeast with no change in substrate specificity or that in *Y. lipolytica*, small 3-OH FAs are more efficiently used by the MFE enzyme than by the PhaC enzyme. Then, we optimized the production of mcl-PHA homopolymer: the addition of a second copy of the 2 enoyl-CoA hydratase gene in *Y. lipolytica* genome, placed under a strong promoter, led to a 1.7-fold increase in polymer accumulation. Overall, the accumulation of both homopolymer and copolymer reached 28% (g/g) of CDW, corresponding to approximatively 2.9 g/L of culture. If we compare this result to the bioproduction of mcl-PHA in other heterologous organisms, our yeast system is more performant. In engineered *Saccharomyces cerevisiae*, mcl-PHA accumulation did not exceed 7% (g/g) of CDW [39], in *Y. lipolytica*, mcl-PHA was produced only at 1 g/L of culture [18] and in the bacteria *Escherichia coli*, mcl-PHA production was estimated to be 6% (g/g) of CDW [24].

In our strategy, we took advantage of the synthesis of 3-OH FAs, as intermediates of the β-oxidation pathway, to produce PHA. Contrary to prokaryotic host in which mcl-PHA synthesis occurs in the cytoplasm, in yeast, the PHA synthesis is confined in the peroxisome where the β-oxidation takes place. This confinement presents a double benefit: it allows the concentration of the substrate (3-OH FA) and the enzyme (PHA synthase), enhancing the production of high molecular weight polymers. In addition, it allows a tight control of the monomeric composition. Indeed, the 3-OH FAs coming from the de novo fatty acid biosynthesis are located in the cytoplasm and cannot be used as substrate for polymer synthesis. Hence, the key interest of our strategy is to take advantage *Y. lipolytica* capacity to efficiently incorporate, activate FA and transport them to the peroxisome where they are degraded or incorporated into PHA.

### Polymer characterizations

The high accumulation rate of both PHA polymers in *Y. lipolytica* allowed us to purify the polymers and to carry out the analysis of their physical, thermal and mechanical properties.

#### Molar masses and purity

The number-average molar mass (M_n_), the mass-average molar mass (M_w_), and the dispersity (Ð) of the PHA samples were determined by size exclusion chromatography (SEC) using THF as eluent. The PHA copolymer showed values of M_n_ and M_w_ around 78,500 and 127,700 g/mol respectively with a dispersity of 1.6 while the PHA homopolymer showed higher M_n_ and M_w_ with values around 156,400 and 316,400 g/mol, respectively, and dispersity of 2 (Table 2). These results indicate a narrow molar mass distribution and M_n_ values similar to commercial P3HB from e.g. Biocycle©, produced by microbial fermentation (M_n_ = 180,000 g/mol and Ð = 2.3, determined by SEC) [40]. Elemental CHN analysis was carried out and showed that the amount of nitrogen, a good indicator of protein content and consequently of purification state, was below 0.5%. This indicates that the amount of residual impurities in the polymers is negligible (Table 2). It is a key point since these impurities are well-known to catalyze the degradation of these biopolymers under thermal input.

**Table 2:** Results of the chains characterizations by SEC and elementary analysis of the PHAs

Sample	SEC			Elementary analysis		
	M_n_ (g/mol)	M_w_ (g/mol)	Ð	C (%)	H (%)	N (%)
PHA copolymer	78,500	127,700	1.6	69.3 ± 0.1	10.4 ± 0.1	0.4 ± 0.03
PHA homopolymer	156,400	316,400	2.0	72.6 ± 0.2	10.9 ± 0.03	0

#### Thermal properties of the mcl-PHAs

Table 3 summarizes the thermal properties of PHAs determined from DSC analyses. As can be seen, the two PHAs presented significant differences in their thermal behaviors. For instance, the lower melting enthalpy (ΔH_m_) and melting point (T_m_), measured for the copolymer indicated a more amorphous behavior than the homopolymer. The glass transition and melting temperature (T_g_ and T_m_) of the PHA polymers were low and indicative of a flexible polymer at ambient temperature. These values are similar with the − 60 °C glass transition temperature and the 60 °C melting temperature of the polycaprolactone a widely used polymer in the field of biomedical application. In addition, although the crystallinity index of these materials could not be calculated since the enthalpies of the perfect PHA crystals are not available for these particular PHAs; a rough estimation of this value was obtained. We used the endothermic melting enthalpy determined by the second heating (ΔH_m_) of each PHA and the melting enthalpy (ΔH_m_^o^) of 100% crystalline P3HB according to equation X_c_ = ΔH_m_/ΔH_m_^o^, assuming 146 J/g as the melting enthalpy of 100% crystalline P3HB as cited in literature [40]. From these results, it is possible to point out that the degree of crystallinity and melting temperature of both PHAs were significantly lower than the commercial P3HB that are 60% and 175 °C, respectively. Additionally, both PHAs presented two melting peaks, possibly due to different crystalline domains.

**Table 3:** Main thermal properties of the PHAs

Sample	DSC cooling				DSC 2nd heating				
	T_c1_ (°C)	T_c2_ (°C)	T_c3_ (°C)	ΔH_c_ (J/g)	T_m1_ (°C)	T_m2_ (°C)	ΔH_m_ (J/g)	Χ_c_ (%)	T_g_ (°C)
PHA copolymer	− 9	n.o	n.o	8.3	19	57	7.7	5.3	− 39
PHA homopolymer	40	5.5	n.o	45.5	45	72	43.9	30.1	n.o

#### Thermogravimetric analysis of the mcl-PHAs

Thermogravimetric analysis (TGA) was used to evaluate the thermal stability of the two PHA (Table 4). Since some PHAs present a high thermal sensitivity such as the P3HB, with chains scissions and polymers degradations, this evaluation is major. The results showed that the polymers were thermally stable up to at least 200 °C and both displayed a single degradation step at a maximum degradation temperature of 225 and 253 °C for the PHA copolymer and the PHA homopolymer, respectively. Published studies reported the highest temperature of maximum degradation rate for a commercial P3HB homopolymer at around 288 °C [40].

**Table 4:** Thermal degradation temperature at 2 wt% loss (T_d,2%_) and maximum degradation (T_d,max_) temperature of the PHAs

Sample	TGA (under nitrogen)	
	T_d,2%_ (°C)	T_d,max_ (°C)
PHA copolymer	204	225
PHA homopolymer	226	253

#### Mechanical properties of the mcl-PHAs

To evaluate the mechanical properties of these two polymers, uniaxial tensile test was performed (Table 5, Additional file 1: Figure S3). The result showed that the PHA copolymer displayed an elastomeric behavior indicated by a low yield stress, low Young’s modulus and high elongation at break values, in agreement with its low melting temperature and low crystallinity. In contrast, the PHA homopolymer showed thermoplastic elastomer properties with high tensile strength, high Young’s modulus and elongation at break values corresponding to a semi-ductile behavior. For comparison, a brittle thermoplastic behavior has been reported for the commercial P3HB from Biocycle© with maximum elongation at break around 4 and 2%, tensile strength around 25 and 40 MPa and flexural modulus of around 3800 and 2200 MPa. The differences observed in the mechanical behavior between the PHAs and the commercial P3HB from Biocycle© agree with their thermal properties.

**Table 5:** Mechanical properties of PHAs under uniaxial tensile tests at room temperature

	Yield stress (MPa)	Young’s modulus (MPa)	Elongation at break (%)
PHA copolymer	4.83 ± 0.65	0.019 ± 0.001	755 ± 151
PHA homopolymer	11.45 ± 2.02	0.410 ± 0.080	347 ± 51

Both polymers displayed interesting properties: the PHA copolymer is an elastomeric material with a low yield stress (4.83 MPa) and a high elongation at break (755%) due to its low melting temperature (two endothermic peaks at around 19 and 57 °C) and low crystallinity (5%). In contrast, the homopolymer has high tensile strength (11.45 MPa) and elongation at break values corresponding to a semi-ductile behavior (347%) due to its low melting temperature (two endothermic peaks at around 45 and 71 °C) and higher crystallinity (30%). Both polymers present lower crystallinity and higher elastomeric properties than P3HB which opens new perspectives of application for PHA.

To summarize the PHA synthesized are amorphous polymers with soft and elastomeric behaviors. Such properties are generally found for medium-chain-length PHAs. It is delicate to compare polymers that do not display equivalent molar masses and that have not been produced in the same conditions. However, we try to briefly confront our biosynthesized PHA to bacterial mcl-PHA previously characterized in the literature. For instance, the T_m_ and T_g_ values measured for our polymers are equivalent and consistent to the values for mcl-PHA produced by *P. putida* (around 60 °C and − 40 °C, respectively) [41–43]. A key-point in this comparison is the static mechanical behavior of our copolymer and, more particularly the elongation at break, higher than 750%. This is one of the highest values reported in the literature for more or less equivalent architectures. Such a behavior can impact directly on potential application areas.

These highly promising biosynthesized materials can find applications in distinct fields. The PHA homopolymer behaves like a soft thermoplastic with a high elongation and a low modulus such as e.g. some low-density polyethylene. Applications can be found for instance on biomedical areas or packaging where the biodegradation is a clear advantage to manage the end-of-life of the corresponding materials. PHA copolymer presents the typical mechanical behavior of a thermoplastic elastomer (TPE) such as some thermoplastic polyurethane (TPU), with uses in biomedical application, shoes, injected rubber gasket, or some seals. However, future studies regarding the mechanical behavior in static or dynamic tests need to be undertaken in connection with these potential functions. In general, the properties of these elastomeric and flexible polymers seem to be adequate for potential biomedical high added values purposes for soft tissues, such as heart valves, cardiac patches and other vascular applications, skin tissue engineering, wound healing and controlled drug release or delivery [14]. However, bioresorbability in contact with living tissues, biocompatibility with in vivo tests and toxicity evaluation have first to be carried out.

#### Future optimization and challenges

The high price of PHA polymer stands as a major drawback for its industrialization. This is due to the use of expensive purified substrate such as glucose and the cost of downstream processes such as extraction and purification. To minimize the overall cost of production, it would be of interest to take advantage of the ability of *Y. lipolytica* to grow on cheaper substrates such as glycerol. Recently, a lot of efforts have been deployed to engineer this yeast to grow on biomass-derived substrates [30, 31]. In addition, optimization of PHA extraction from eukaryotic microorganism will have to be investigated. In the case of oleaginous organism, recent advances have been made to facilitate the process of lipid extraction and could be extended to PHA extraction [44, 45].

## Conclusion

This study presents the optimization of the bioproduction of mcl-PHA polymers. We showed that metabolic engineering of the yeast *Y*. *lipolytica* lead to the production of two types of mcl-PHA in a heterologous microorganism at levels never reached before. This allowed us to fully investigate the biophysical properties of these polymers and to extrapolate on their potential applications. This work clearly describes a successful pluridisciplinary approach that opens interesting opportunities for producing custom made polymers and highlights the power of engineering to generate materials meeting the needs of society for a more sustainable future.

## Additional file

**Additional file 1: Table S1.** Strains used in this study. pTEF-PhaC: PhaC synthase from Pseudomonas aeruginosa targeted to peroxisome and expressed under the promoter pTEF; wt: wild type; pPOX2-MFE: MFE enzyme from *Y. lipolytica* expressed under the promoter pPOX2. MFEABC, the whole protein with its three catalytic domains is expressed; MFEC: only the domain C (encoding the 2 enoyl hydratase) is expressed. **Table S2.** polymer accumulation and composition in *Y. lipolytica* strains expressing different variants of phaC synthase. **Table S3.** homopolymer accumulation in *Y. lipolytica* strains expressing the MFEC domain and grown with different source of fatty acids as substrate. **Table S4.** Sequence of the primers used in directed site mutagenesis on PhaC. **Figure S1.** ^1^H NMR spectrum in CDCl_3_ with 1% TMS of the cellular extracts of strain ThYl 1166, grown in YNB supplemented with mC14 showing almost exclusively signals belonging to a mcl-PHA, and traces of free FA. **Figure S2.** ^1^H NMR spectrum in CDCl_3_ with 1% TMS of the cellular extracts of strain ThYl 1024, grown in YNB supplemented with C12 showing almost exclusively signals belonging to a homopolymer of 3HDD and traces of free FA. **Figure S3.** Stress-strain curves of synthesized PHAs stored at 37 °C during 5 days.

## Data Availability

The datasets used during the current study are available from the corresponding author on reasonable request.

## Abbreviations

PHA: polyhydroxyalkanoate
scl-PHA: short-chain-length polyhydroxyalkanoate
mcl-PHA: medium-chain-length polyhydroxyalkanoate
P3HB: poly-3-hydroxybutyrate
FA: fatty acid
3-OH FA: 3-hydroxy fatty acid
3HO: 3-hydroxyoctanoic acid
3HD: 3-hydroxydecanoic acid
3HDD: 3-hydroxydodecanoic acid
3TD: 3-hydroxytetradecanoic acid
