# Supplementary material for: Production and characterization of two medium-chain-length polydroxyalkanoates by engineered strains of Yarrowia lipolytica
Source: Microb Cell Fact. 2019 May 31;18:99. doi: 10.1186/s12934-019-1140-y (PMC6545009; doi:10.1186/s12934-019-1140-y)
Supplement: Supplementary file 1 — Additional file 1: Table S1. Strains used in this study. pTEF-PhaC: PhaC synthase from Pseudomonas aeruginosa targeted to peroxisome and expressed under the promoter pTEF; wt: wild type; pPOX2-MFE: MFE enzyme from Y. lipolytica expressed under the promoter pPOX2. MFEABC, the whole protein with its three catalytic domains is expressed; MFEC: only the domain C (encoding the 2 enoyl hydratase) is expressed. Table S2. polymer accumulation and composition in Y. lipolytica strains expressing different variants of phaC synthase. Table S3. homopolymer accumulation in Y. lipolytica strains expressing the MFEC domain and grown with different source of fatty acids as substrate. Table S4. Sequence of the primers used in directed site mutagenesis on PhaC. Figure S1. 1H NMR spectrum in CDCl3 with 1% TMS of the cellular extracts of strain ThYl 1166, grown in YNB supplemented with mC14 showing almost exclusively signals belonging to a mcl-PHA, and traces of free FA. Figure S2. 1H NMR spectrum in CDCl3 with 1% TMS of the cellular extracts of strain ThYl 1024, grown in YNB supplemented with C12 showing almost exclusively signals belonging to a homopolymer of 3HDD and traces of free FA. Figure S3. Stress-strain curves of synthesized PHAs stored at 37 °C during 5 days. [file 12934_2019_1140_MOESM1_ESM.docx]

**Production and characterization of two medium chain length polydroxyalkanoates by engineered strains of *Yarrowia lipolytica***

Coraline Rigouin^1,4^, Sophie Lajus^1,4^, Connie Ocando^2,4^, Vinciane Borsenberger^1^, Jean Marc Nicaud^3^, Alain Marty^1^, Luc Avérous^2^, Florence Bordes^1^,

^1^ LISBP, Université de Toulouse, CNRS, INRA, INSA, Toulouse, France

^2^ BioTeam/ICPEES-ECPM, UMR CNRS 7515, Université de Strasbourg, 25 rue Becquerel, 67087 Strasbourg Cedex 2, France

^3^ Micalis Institute, INRA-AgroParisTech, UMR1319, Team BIMLip: Integrative Metabolism of Microbial Lipids, Domaine de Vilvert, 78352 Jouy-en-Josas, France.

^4^ These authors contributed equally to this work

* Corresponding author: [bordes@insa-toulouse.fr](mailto:bordes@insa-toulouse.fr)

| **Strain name** | **Genotype** | **Source or reference** |
| --- | --- | --- |
| **JMY_1877** | Q4= MATA ura3-302 leu2-270 xpr2-322 ∆dga1 ∆lro1 ∆dga2 ∆are1 | Beopoulos et al, 2012 |
| **JMY1915** | Q4 ∆mfe1 | Haddouche *et al*., 2011 |
| **JMY2333** | Q4 Δmfe1 pPOX2-MFEABC | This work |
| **JMY2475** | Q4 Δmfe1 pPOX2-MFEC | This work |
| **ThYl_1475** | Q4, pTEF-PhaCwt-URA3ex, LEU2ex | This work |
| **ThYl_1479** | Q4, pTEF-PhaC_E130D-URA3ex, LEU2ex | This work |
| **ThYl_1480** | Q4, pTEF-PhaC_E130D S477R Q481M-URA3ex, LEU2ex | This work |
| **ThYl_1481** | Q4, pTEF-PhaC_E130D S477F Q481K-URA3ex, LEU2ex | This work |
| **ThYl_1485** | Q4, pTEF-PhaC_A547V-URA3ex, LEU2ex | This work |
| **ThYl_1487** | Q4, pTEF-PhaC_S482G L484V-URA3ex, LEU2ex | This work |
| **ThYl_1491** | Q4, pTEF-PhaC_E130D S325T S477R Q481M S482G L484V-URA3ex, LEU2ex | This work |
| **ThYl_1494** | Q4, pTEF-PhaC_E130D S325T S477R Q481M-URA3ex, LEU2ex | This work |
| **ThYl_1496** | Q4, pTEF-PhaC_E130D S325T S477R Q481M A547V-URA3ex, LEU2ex | This work |
| **ThYl_1498** | Q4, pTEF-PhaC_E130D S325T S477R Q481M A547V S482G L484V-URA3ex, LEU2ex | This work |
| **ThYl_1166** | Q4, Δmfe1, pPOX2-MFEABC, pTEF-PhaC_E130D S325T S477R Q481M-URA3ex, pTEF-CpPCT-LEU2ex | This work |
| **ThYl_657** | Q4, Δmfe1, pPOX2-MFEC, pTEF-PhaC_E130D S325T S477R Q481M-URA3ex, LEU2ex | This work |
| **ThYl_1024** | Q4, Δmfe1, pPOX2-MFEC, 4UAS-MfeC, pTEF-PhaC_E130D S325T S477R Q481M-URA3ex, LEU2ex | This work |

**Table S1**: Strains used in this study. pTEF-PhaC: PhaC synthase from Pseudomonas aeruginosa targeted to peroxisome and expressed under the promoter pTEF; wt: wild type; pPOX2-MFE: MFE enzyme from *Y. lipolytica* expressed under the promoter pPOX2. MFEABC, the whole protein with its three catalytic domains is expressed; MFEC: only the domain C (encoding the 2 enoyl hydratase) is expressed.

|  | |  | **Fraction of 3-OH FA (mmol)** | | | |
| --- | --- | --- | --- | --- | --- | --- |
| **Strain name** | **Polymer accumulation % (g/g)** | | **3HO** | **3HD** | **3HDD** | **3HTD** |
| ThYl_1475 | 7 | | 30% | 35% | 26% | 9% |
| ThYl_1479 | 13 | | 27% | 35% | 28% | 11% |
| ThYl_1480 | 11 | | 30% | 32% | 26% | 13% |
| ThYl_1481 | 10 | | 32% | 33% | 25% | 10% |
| ThYl_1485 | 9 | | 29% | 35% | 26% | 10% |
| ThYl_1487 | 10 | | 29% | 33% | 28% | 9% |
| ThYl_1491 | 28 | | 26% | 35% | 29% | 10% |
| ThYl_1494 | 27 | | 29% | 36% | 27% | 9% |
| ThYl_1496 | 0 | | / | / | / | / |
| ThYl_1498 | 6 | | 35% | 36% | 22% | 8% |

**Table S2**: polymer accumulation and composition in *Y. lipolytica* strains expressing different variants of phaC synthase.

| **Strain name** | **Fatty acid** | **Polymer accumulation % (g/g)** |  |
| --- | --- | --- | --- |
| ThYl_657 | mC12 | 9 |  |
| ThYl_657 | mC14 | 2 |  |
| ThYl_1024 | mC12 | 15 |  |
| ThYl_1024 | mC14 | 4 |  |

**Table S3:** homopolymer accumulation in *Y. lipolytica* ***s***trains expressing the MFEC domain and grown with different source of fatty acids as substrate.

|  | |
| --- | --- |
| **Sequence 5’ - 3’** | **Mutations** |
| CAACCTGCTCACCGACGCTATGTCTCCCACC | E130D |
| GGTGGGAGACATAGCGTCGGTGAGCAGGTTG | E130D |
| CCCAGCTGGTGACCGTGCTGGACTTTGAGC | S325T |
| GCTCAAAGTCCAGCACGGTCACCAGCTGGG | S325T |
| CCTGTCCAACCGAGGCCACATCATGTCTATCCTGAACC | S477R and Q481M |
| GGTTCAGGATAGACATGATGTGGCCTCGGTTGGACAGG | S477R and Q481M |
| CCTGTCCAACTTCGGCCACATCAAGTCTATCCTGAACC | S477F and Q481K |
| GGTTCAGGATAGACTTGATGTGGCCGAAGTTGGACAGG | S477F and Q481K |
| GCAACAAGACCTACCCCGTCGGCGAGGCCGCTCC | A547V |
| GGAGCGGCCTCGCCGACGGGGTAGGTCTTGTTGC | A547V |
| GAGGCCACATCATGGGTATCGTCAACCCTCCCGGCAAC | S482G and L484V |
| GTTGCCGGGAGGGTTGACGATACCCATGATGTGGCCTC | S482G and L484V |

**Table S4:** Sequence of the primers used in directed site mutagenesis on PhaC

**
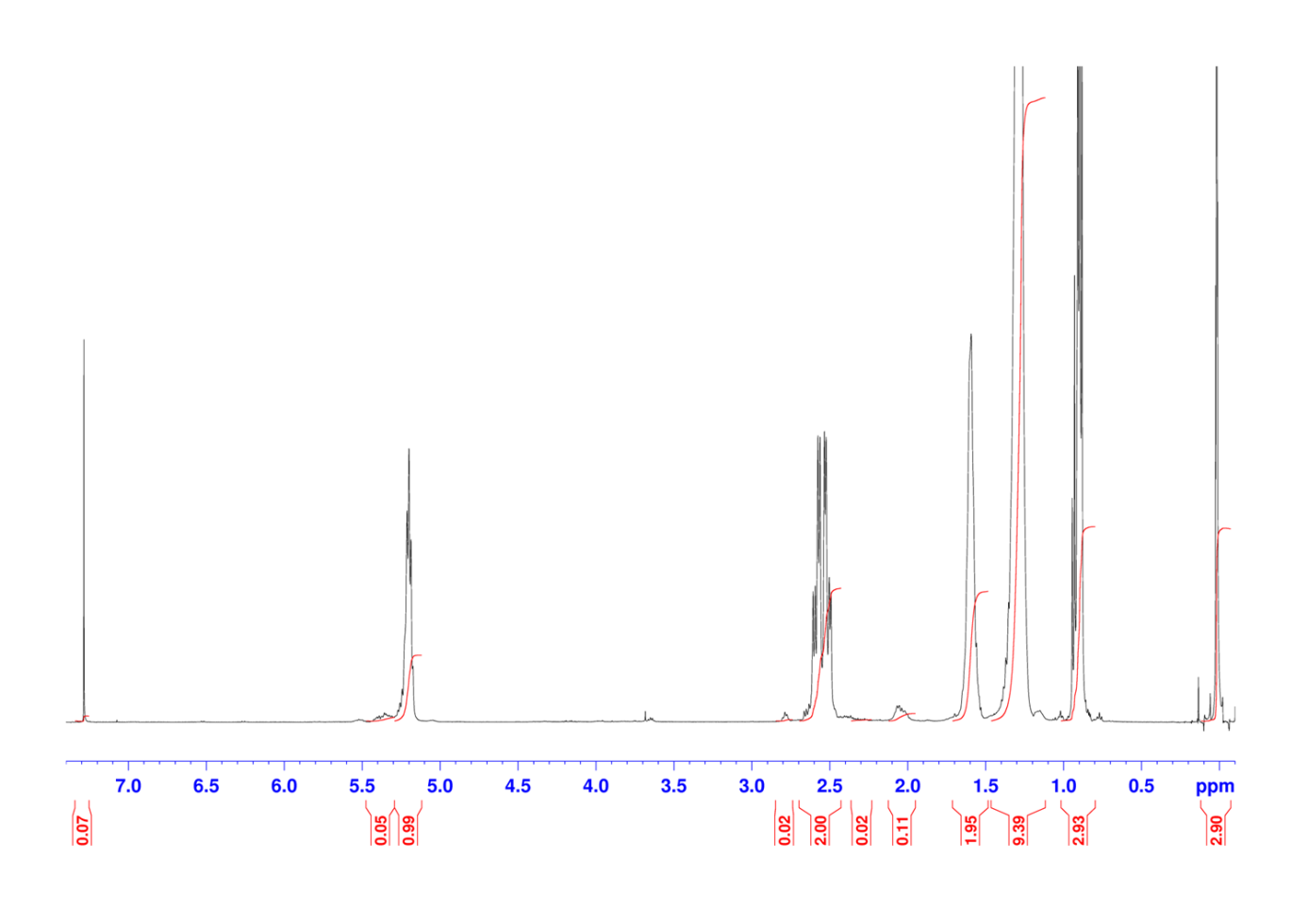
**

**Figure S1**: ^1^H NMR spectrum in CDCl_3_ with 1% TMS of the cellular extracts of strain ThYl 1166, grown in YNB supplemented with mC14 showing almost exclusively signals belonging to a mcl-PHA, and traces of free FA.

**
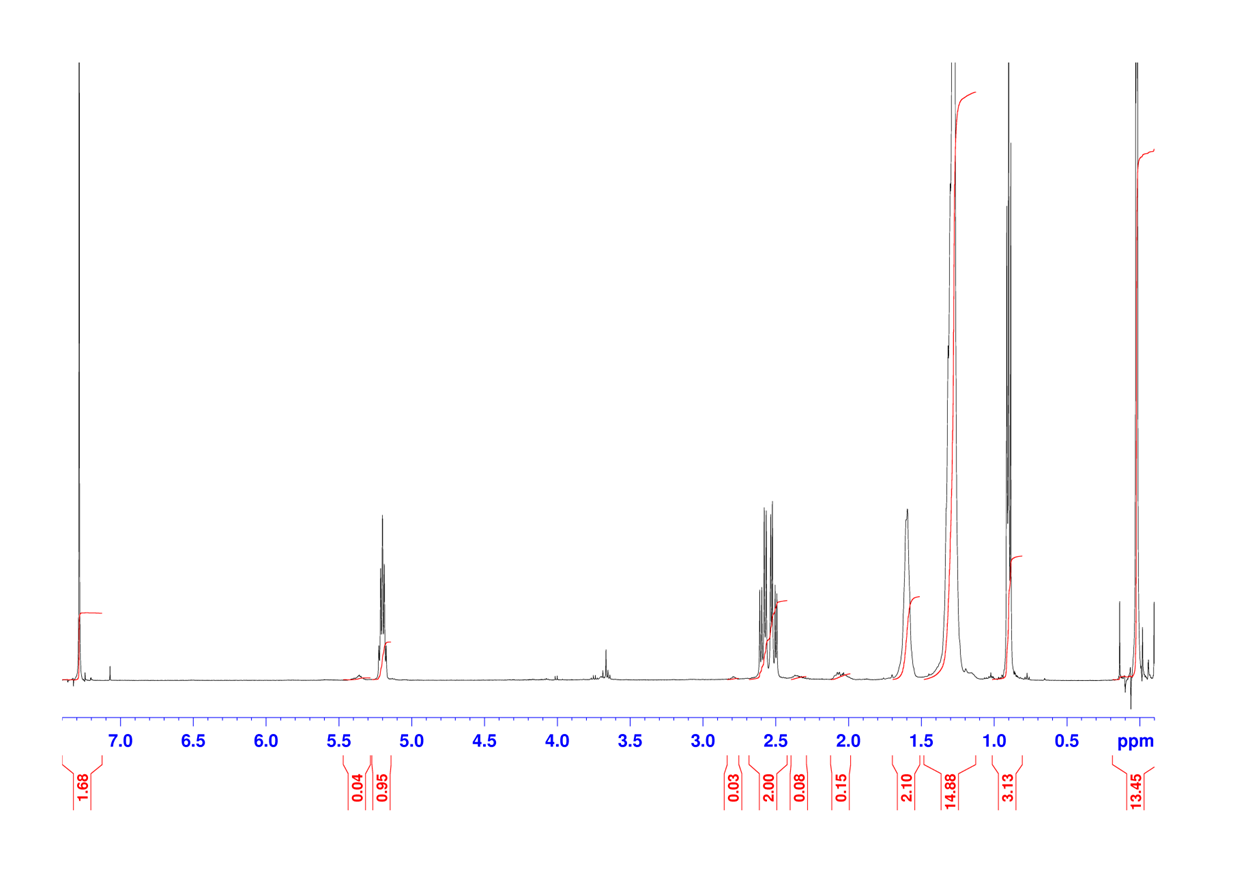
**

**Figure S2**^: 1^H NMR spectrum in CDCl_3_ with 1% TMS of the cellular extracts of strain ThYl 1024, grown in YNB supplemented with C12 showing almost exclusively signals belonging to a homopolymer of 3HDD and traces of free FA.

**
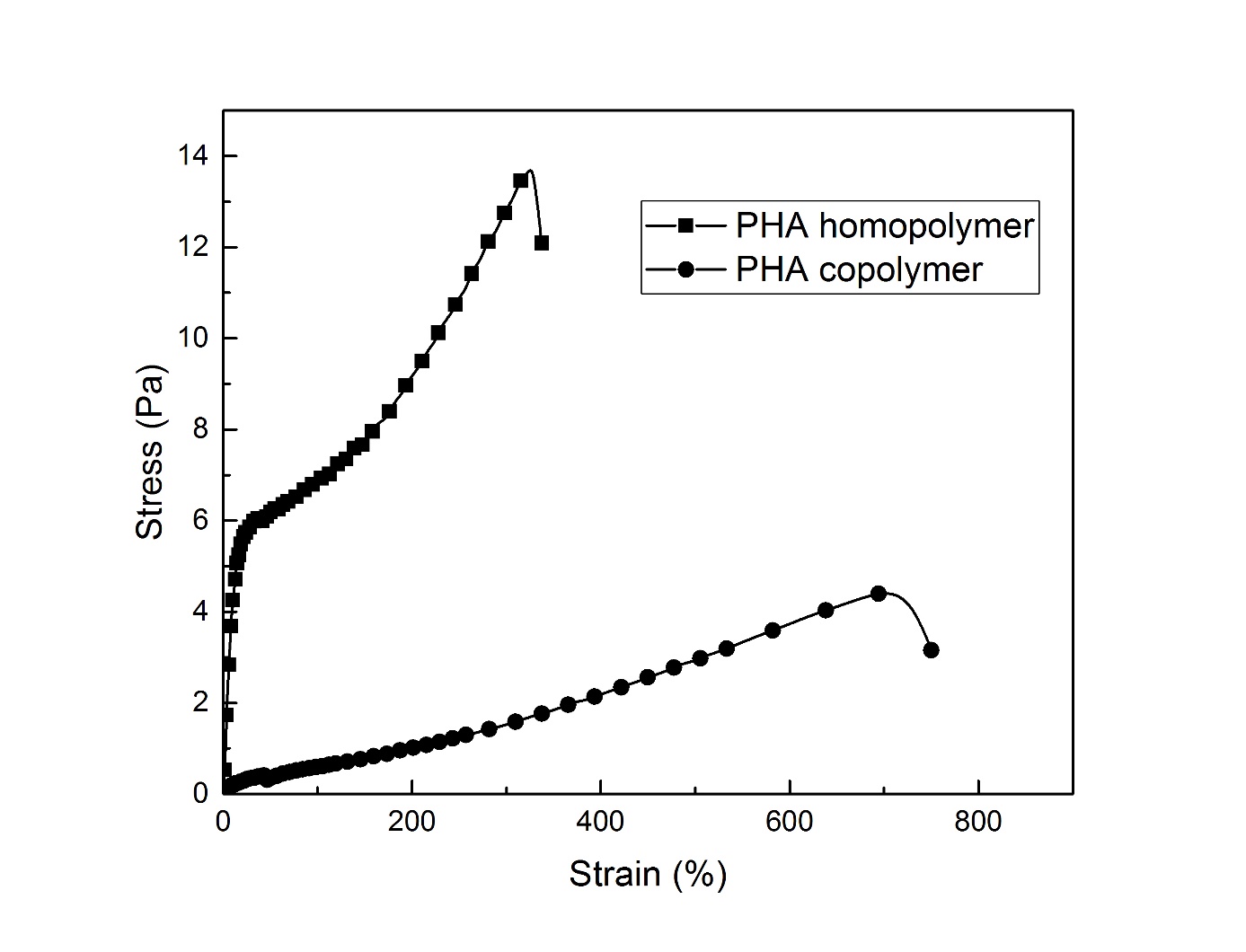
**

**Figure S3:** Stress-strain curves of synthesized PHAs stored at 37 °C during 5 days
